# Construction of an HRP-streptavidin bound antigen and its application in an ELISA for porcine circovirus 2 antibodies

**DOI:** 10.1186/s13568-017-0473-3

**Published:** 2017-09-18

**Authors:** Meng Ge, Run-Cheng Li, Tailong Qu, Wenjie Gong, Xing-Long Yu, Changchun Tu

**Affiliations:** 1 Institute of Military Veterinary Medicine, Academy of Military Medical Sciences, No. 666 Liuying Xi Road, Changchun, 130122 Jilin People’s Republic of China; 2 College of Veterinary Medicine, Hunan Agricultural University, No. 1 Nongda Road, Changsha, 410128 Hunan People’s Republic of China

**Keywords:** HRP-streptavidin bound antigen, Double-antigen sandwich ELISA, PCV2 antibodies

## Abstract

A fusion protein SBP-Cap∆41, consisting of Cap∆41 (without 41 amino acids at the N-terminus) protein of porcine circovirus 2 (PCV2) and a streptavidin binding peptide (SBP), was constructed. This fusion protein binds to HRP-labeled streptavidin (HRP-SA) through high affinity between SBP and SA, forming an HRP-streptavidin bound antigen (Hsb-Ag) with both immunoreactivity and enzymatic activity, which can be used in a double-antigen sandwich ELISA for detection of PCV2 antibodies. Comparison of the characteristics of the HSb-Cap∆41 and chemical conjugates of the recombinant Cap∆41 protein showed that the HSb-Cap∆41 based double-antigen sandwich ELISA (HBDS-ELISA) had higher specificity and sensitivity. Use of the HBDS-ELISA detected PCV2-IgG in 9 injected pigs as early as 10 days p.i., 3 days earlier than both a double-antigen sandwich ELISA (DS-ELISA) based on a chemically conjugated antigen, and a commercial indirect ELISA kit.

**Electronic supplementary material:**

The online version of this article (doi:10.1186/s13568-017-0473-3) contains supplementary material, which is available to authorized users.

## Introduction

PCV2 is a small non-enveloped icosahedral virus with a single-stranded circular DNA genome (Khayat et al. 2011) that poses a serious hazard to pig health and causes great economic losses in the pig-raising industry (Chae 2005; Segalés 2012). We have previously reported a double-antigen sandwich ELISA (DS-ELISA) for PCV2 antibody detection using two HRP-conjugated antigens, HRP-Trx-Cap∆41 and HRP-Cap∆41 (Ge et al. 2012). The sensitivity of the HRP-Cap∆41-based ELISA was lower than HRP-Trx-Cap∆41 due to modification of epitopes on the Cap∆41 protein during HRP conjugation, resulting in loss of specific binding activity to serum PCV2 antibody. Conjugation of HRP to Trx may therefore have spared some important Cap∆41 epitopes in Trx-Cap∆41 conjugates.

Conjugated antigens, connecting the antigen–antibody reaction with the enzymatic reaction, are the most important elements in double-antigen sandwich ELISAs. Generally, such antigens, prepared by traditional chemical methods based on formation of covalent bonds between reactive groups of proteins and bifunctional agents may result in loss of immunogenic or other activity (Porstmann and Kiessig 1992; Lequin 2005; Hermanson 2008). Conventional conjugation methods generally work well with antibodies (Wilson and Nakane 1978); however, results with many antigens of less well-established structures are less constant (Hermanson 2008; Ge et al. 2012). This is probably the main reason why double-antigen sandwich ELISAs are not as commonly used for antibody detection as indirect ELISA and blocking ELISA.

**Fig. 1:**
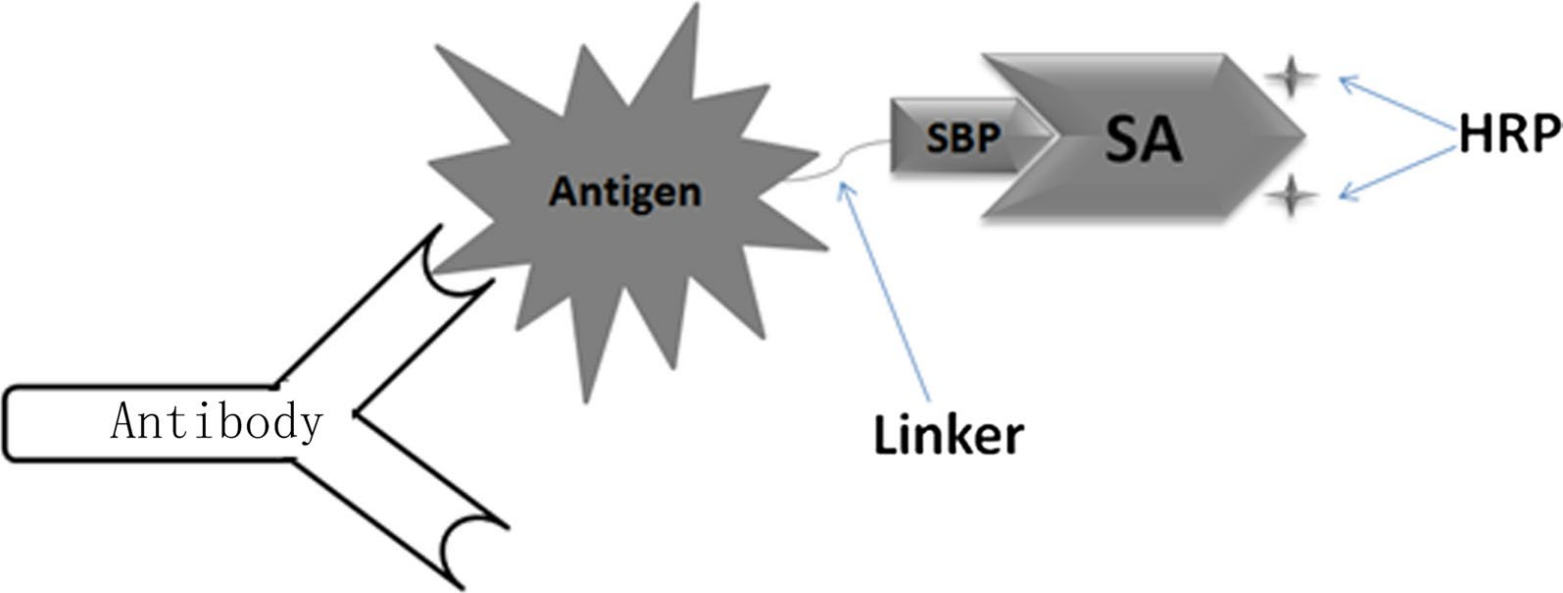
Hsb-Ag (shown in gray) and its binding to antibody. SBP is fused to antigen by a linker. The recombinant antigen is able to bind to HRP labeled streptavidin (SA) with high affinity and retention of reactionogenicity. In this report, the antigen and specific antibody are Cap∆41 and PCV2 antibody, respectively

To circumvent the effect on antigenic structure caused by conventional chemical conjugation that could result in decrease in sensitivity of DS-ELISAs, a novel HRP-conjugated antigen (Cap∆41) was constructed by affinity binding (Fig. 1) and utilized to establish a new double-antigen sandwich ELISA for PCV2 antibody detection.

## Materials and methods

### Construction of the recombinant plasmid encoding SBP-Cap∆41 fusion protein

The SBP gene with a GS linker (coding amino acid sequence: GGGGSGGGGSGGGGS) and a 6× His-Tag was obtained by polymerase chain reaction (PCR) with the pBEn-SBPa vector (Stratagene, USA) as a template. The primer sequences for the PCR were 5′-TACCATGGGCTGCAAGCTGGGCCTG-3′ (upstream) and 5′-TGAATTCCGAGCCGCCACCACCTGAACCGCCACCACCCGAGCCACCACCGCCGTGATGATGATGATGGTGCAGGCCCAGCTTGCAGC-3′ (downstream). The PCR products were double digested with *Nco*I and *Eco*RI and cloned into pET-28a(+) vector (Novagen, USA), producing pET-28S. The PCV2 Cap∆41 protein (capsid protein without 41-amino acid [aa] N-termina nuclear localization signal peptide) gene was amplified using the genome of PCV2 strain YZH (GenBank Accession Number AY943819) as template according to previously study (Ge et al. 2012). The PCR products were double digested with *Eco*RI and *Sal*I and cloned into pET-28S, producing pET28S-Cap∆41. The construct was verified by DNA sequencing (GenScript, Nanjing, China).

### SBP-Cap∆41 fusion protein expression and purification

For expression of SBP-Cap∆41 fusion protein, Rosetta (DE3) pLysS *E. coli* cells (Novagen, USA) were transfected with plasmid pET28S-Cap∆41. A single colony of transformants was cultivated in Luria–Bertani medium in an incubator shaker at 37 °C to an optical density of 0.6 at 600 nm. Isopropylthio-β-d-galactopyranoside (IPTG) was added to a final concentration of 1 mM. After induction at 30 °C for 6 h, cells were harvested by centrifugation. Purification of the expressed SBP-Cap∆41 fusion protein was by immobilized metal affinity chromatography using the His-Bind Purification kit (Novagen, USA) according to the manufacturer’s instructions.

### Preparation of HRP-streptavidin bound Cap∆41 (Hsb-Cap∆41)

The Hsb-Cap∆41 was constructed by simply mixing HRP-SA (Pierce, USA; ~7.0 × 10^−8^ M) and SBP-Cap∆41 (2.8 × 10^−7^ M) in equal volumes, and incubating for 48 h at 4 °C.

### Reactivity between the Hsb-Cap∆41 and PCV2 serum antibodies

An immune assay was performed to determine whether the Hsb-Cap∆41 had specific reactivity to PCV2 antibody. The recombinant Cap∆41 protein was prepared according to the method described in the previous study (Ge et al. 2012) and diluted in 0.05 M NaHCO_3_/Na_2_CO_3_ buffer (pH 9.6). The wells of high binding 96-well microtitration plates (Costar, Corning, NY, USA) were coated with 100 µl Cap∆41 protein (100 ng/well) at 4 °C for 24 h. After incubation, the wells were washed 3 times with PBST and blocked with 250 µl 5% dried skim milk in 0.01 mM PBST (pH 7.4) at 37 °C for 2 h. Following three washes with PBST, the plates were dried at room temperature. Eight serum samples (four positive and four negative) were diluted 1:9 with PBST, and 100 µl of each dilution was added to the microtitration plate wells. After incubation for 60 min at 37 °C, followed by five rounds of washing with PBST, 100 µl aliquots of Hsb-Cap∆41 diluted 1/10, 1/100 or 1/1000 were then added. After a further incubation of 60 min at 37 °C, followed by five rounds of washing with PBST, 50 µl/well of 3,3′,5,5′-tetramethylethylenediamine solution (SureBlue Reserve TMB Microwell Peroxidase Substrate, KPL) was added and the plates were incubated for 15 min at 37 °C. The chromogenic reaction was stopped by the addition of 50 µl 2 M sulfuric acid, and the optical density at 450 nm (OD_450_) was recorded using a microplate reader (MK3; Thermo Lab system, Helsinki, Finland).

### Double-antigen sandwich ELISA based on Hsb-Cap∆41

The Hsb-Cap∆41 based double-antigen sandwich ELISA (HBDS-ELISA) was developed according to the method described in the previous study (Ge et al. 2012). All the conditions of the two ELISAs were kept the same except for the HRP-conjugated antigen. The detailed process was as follows: Cap∆41 protein coated plates were prepared as described above. Hsb-Cap∆41 was serially diluted twofold from 1:25 to 1:400 in PBST. Each dilution was mixed with positive and negative control serum in ratios of 1:9, and then 100 µl aliquots of the mixtures were added to the microtitration plate wells. After incubation for 60 min at 37 °C, followed by five rounds of washing with PBST, the chromogenic reaction and the following steps were as described above. The dilution of Hsb-Cap∆41 with the highest P/N ratios (positive control OD_450_/negative control OD_450_) and the OD_450_ value of the positive serum closest to 1.0 were considered optimal.

To confirm the negative–positive cutoff value for the HBDS-ELISA, 60 serum samples collected sequentially from 12 PCV-free pigs testing negative for PCV2 antibody by DS-ELISA and a commercial indicated ELISA Kit (JENO Biotech Inc, Korea) were tested using the HBDS-ELISA. Antibody titers of the samples were calculated according to the formula:

$${\text{S}}/{\text{P}} = \left( {{\text{sample OD}}_{ 450} - {\text{negative control OD}}_{ 450} } \right)/\left( {{\text{positive control OD}}_{ 450} - {\text{negative control OD}}_{ 450} } \right).$$

Mean S/P (X) and standard deviations (SD) of the 60 negative sera were calculated, and the negative–positive cutoff value was determined as X + 3SD.

### Reproducibility of the HBDS-ELISA

Twelve HBDS-ELISA positive and 12 HBDS-ELISA negative field serum samples were selected to evaluate the reproducibility of the assay and the procedure was performed as proposed by Jacobson (1998). For intra-assay (within plate) reproducibility, three replicates of each serum sample were assigned in the same plate. For interassay (between run) reproducibility, three replicates of each sample were run in different plates. Mean S/P ratio, standard deviation (SD) and coefficient of variation (CV) were calculated.

### Performance evaluation of the HBDS-ELISA

Both DS-ELISA and the commercial indirect ELISA kit were used as the reference methods to evaluate the HBDS-ELISA. A total of 269 serum samples were used to compare the three methods. Of these, 60 were the negative sera as described above. Ninety-two sera were selected at random from field samples testing positive by DS-ELISA. Sixty-three samples were collected every 4 weeks from nine piglets ranging from 4 to 28 weeks old in a PCV2 infected farm. The remaining 54 samples came from nine PCV-free pigs injected with PCV2 and collected at 0, 3, 7, 10, 13 and 16 days p.i. The diagnostic sensitivity (DSN) and specificity (DSP) were calculated as follows: ${\text{DSN}} = {\text{TP}}/\left( {{\text{TP}} + {\text{FN}}} \right) \times 100,$ ${\text{DSP}} = {\text{TN}}/\left( {{\text{TN}} + {\text{FP}}} \right) \times 100,$ where TP, FN, TN and FP represent true-positive, false-negative, true-negative and false-positive, respectively (Jacobson 1998). The kappa statistic was used to measure the strength of agreement between the results of the HBDS-ELISA and the reference methods. A kappa statistic of >0.75 represents excellent agreement, 0.40–0.75, good to fair agreement, and <0.40, poor agreement (Pottumarthy et al. 1999).

For further comparison of the three methods, the overall dynamics of serum PCV2 antibody production in the nine naturally infected and nine experimentally infected pigs were analyzed. Additionally, to check for possible cross-reactivity of the HBDS-ELISA with a view to confirming its specificity, positive sera of classic swine fever virus (CSFV), porcine reproductive and respiratory syndrome virus (PRRSV), porcine pseudorabies virus (PRV), and porcine parvovirus (PPV) from PCV-free pigs were tested according to the HBDS-ELISA procedure.

## Results

### Expression and purification of the recombinant protein

**Fig. 2:**
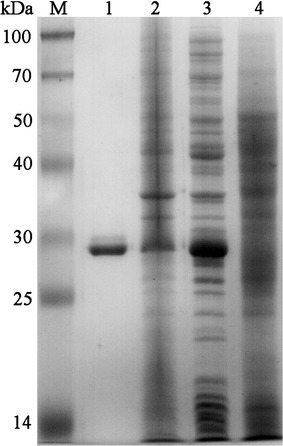
SDS-PAGE analysis of expressed and affinity column purified SBP-Cap∆41 protein. *M* protein marker; *1* purified SBP-Cap∆41 protein; *2* pellet of cell lysate after sonication and centrifugation; *3* supernatant of cell lysate after sonication and centrifugation; *4* lysates of pET-28a(+) expressing cells

According to the result of DNA sequencing, the recombinant plasmids pET28S-Cap∆41 was successfully constructed and the sequence of Cap∆41 (Additional file 1: Text S1) showed 100% identity with genome sequence of PCV2 strain YZH. As determined by SDS-PAGE analysis, the recombinant fusion protein SBP-Cap∆41 was expressed in soluble as well as in inclusion body form of approximately 29 kDa (Fig. 2); i.e., the theoretical molecular weight of the fusion proteins. The soluble form was used in the subsequent construction of the Hsb-Cap∆41 and development of the HBDS-ELISA.

### Reactivity between Hsb-Cap∆41 and PCV2 antibodies

**Fig. 3:**
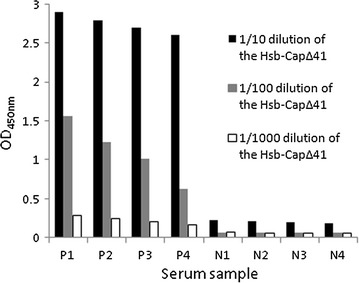
Reactivity of Hsb-Cap∆41 with PCV2 positive and PCV2 negative sera. *P1*–*P4* 4 PCV2 positive serum samples. *N1*–*N4* 4 PCV2 negative serum samples

Immunoassay showed that the Hsb-Cap∆41 reacted with PCV2 positive but not PCV2 negative serum samples. This indicated that the Hsb-Cap∆41 had specific reactivity with PCV2 antibodies. The working optimal dilution of the Hsb-Cap∆41 was approximately 1:100 and the background was low (Fig. 3).

### Working conditions and cutoff value of the HBDS-ELISA

The optimal dilution of Hsb-Cap∆41, 1:50, gave an OD_450_ value of ~1.0 for the positive control serum and a highest P/N ratio of 12.07 ± 0.041. The other conditions of the HBDS-ELISA were the same as DS-ELISA described previously (Ge et al. 2012).

The mean S/P ratios (X) and standard deviations (SD) of the 60 negative sera were 0.052 and 0.049, respectively, giving a negative–positive cutoff S/P value of 0.20.

### Reproducibility

The reproducibility of the HBDS-ELISA was determined by comparing S/P ratios of each serum sample. The intra-assay coefficient of variation (CV) of the 12 positive serum samples tested ranged from 1.97 to 7.35%, (median 4.86%), and interassay CV from 2.98 to 11.58% (median 7.16%). In addition, the HBDS-ELISA also showed good reproducibility for 12 negative field sera (data not shown).

### Performance evaluation

**Fig. 4:**
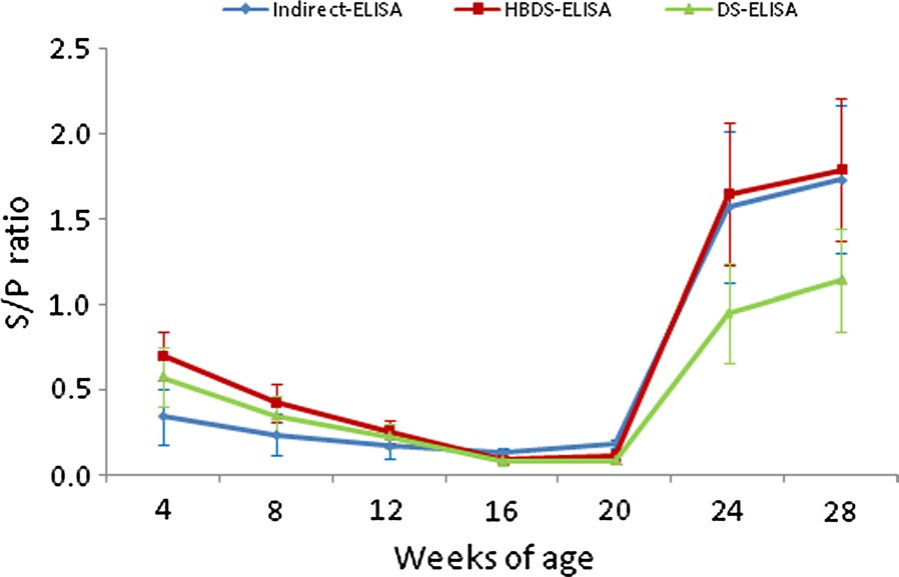
Mean PCV2 specific serum antibody titers of the nine naturally infected with PCV2. Standard deviations are indicated by the error bars. The S/P cutoffs of the HBDS-ELISA, DS-ELISA and Indirect ELISA were 0.2, 0.27 and 0.35, respectively

**Fig. 5:**
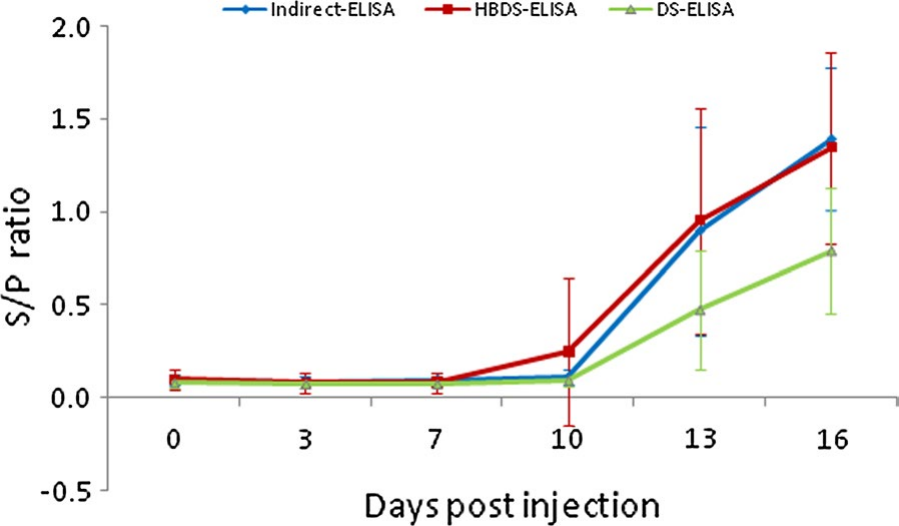
Mean PCV2 specific serum antibody titers of the nine pigs experimentally infected with PCV2. Standard deviations are indicated by the error bars. The S/P cutoffs of the HBDS-ELISA, DS-ELISA and Indirect ELISA were 0.2, 0.27 and 0.35, respectively

The results of all the 269 serum samples determined by the three methods are shown in Table 1. The HBDS-ELISA showed good consistency with DS-ELISA (kappa value 0.918) and indirect ELISA (kappa value 0.895), with DSN and DSPs of 100 and 91.47% for DS-ELISA, and 100 and 88.72% for the commercial kit respectively. The dynamics of serum PCV2 antibody production in the nine naturally infected pigs and the nine experimentally infected pigs are shown in Figs. 4, 5, respectively. The sensitivity of the HBDS-ELISA was higher for detection of PCV2 antibodies than the other two assays. For the nine naturally infected pigs, the S/P ratios of the positive sera were higher by HBDS-ELISA than that by DS-ELISA at most of stages during the experimental period. Compared with indirect ELISA, the S/P ratios of positive sera were higher by HBDS-ELISA, especially before seroconversion (Fig. 4). In the nine experimentally infected pigs, PCV2 antibodies were detected as early as 10 days p.i., 3 days earlier than the DS- and indirect ELISAs (Fig. 5).

**Table 1:** Comparison of the HBDS-ELISA with the DS-ELISA and the commercial indirect ELISA kit

HBDS-ELISA^a^	No. of serum samples					
	DS-ELISA^b^			Indirect ELISA^c^		
	Positive	Negative	Total	Positive	Negative	Total
Positive	140	11	151	136	15	151
Negative	0	118	118	0	118	118
Total	140	129	269	136	133	269

^a^S/P ratio cutoffs: positive ≥0.20; negative <0.20

^b^S/P ratio cutoffs: positive ≥0.27; negative <0.27

^c^S/P ratio cutoffs: positive ≥0.35; negative <0.35

To test for cross-reactivity of the HBDS-ELISA with antibodies against other porcine viruses, four serum samples positive for CSFV, PRRSV, PRV or PPV were tested in triplicate. The S/P ratios of all these sera were <0.1 (Table 2), showing that there was no significant cross-reaction.

**Table 2:** Cross-reactivity analysis of the HBDS-ELISA against other swine viral antisera

Virus antiserum	OD_450_ (±SD)	S/P ratio^a^
CSFV	0.069 ± 0.006	0.025
PRRSV	0.101 ± 0.004	0.059
PRV	0.093 ± 0.003	0.051
PPV	0.089 ± 0.002	0.046

^a^S/P ratio cutoffs: positive ≥0.20; negative <0.20

## Discussion

The double-antigen sandwich ELISA is a method for antibody detection. In this ELISA system, serum specific IgG antibody binds to the antigen in the solid phase by one of two binding sites and capture conjugated antigen in the liquid phase by the other (Miyazawa et al. 1988, 1999). It generally exhibits high specificity because the conjugated antigen binds only to specific IgG antibody. Serum samples can be added without dilution (He et al. 2011; Venteo et al. 2012), which increases the sensitivity and simplifies the procedure. Another advantage is that it is species non-specific which makes it particularly useful for seroepidemiologic surveys of zoonoses (Hu et al. 2008; Watcharatanyatip et al. 2010). Although the double-antigen sandwich ELISA has its advantages, the applicability has been limited because of modifications to the antigenic structure caused by chemical conjugation. The technique described here largely circumvents this drawback.

Like biotin, streptavidin binding peptide (SBP) has a binding affinity to streptavidin (SA), and this property was exploited to fuse it to the antigen molecule. The resulting fusion protein binds to the HRP-labeled streptavidin (HRP-SA) with high affinity, forming an HRP-conjugated antigen complex with both antigenicity and enzymatic activity. By avoiding conjugating the HRP to antigen directly, the diagnostic sensitivity is improved since the structure of the antigen is less affected and steric hindrance between antigen and antibody is decreased. By non-competitive ELISA the affinity constant Ka of the reaction between SBP-Cap∆41 and HRP-SA was found to be 8.3 × 10^8^ M^−1^; i.e., higher than that of most immune reactions between antigens and antibodies (Braden and Poljak 1995; Loomans et al. 1995; Zheng et al. 2004).

**Fig. 6:**
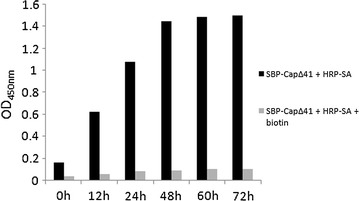
Effect of biotin on measurement of antibody by HBDS-ELISA. The biotin was added to a final concentration of 2.8 × 10^−7^ M; i.e., equimolar with SBP-Cap∆41

In order for the fusion protein SBP-Cap∆41 to bind to both HRP-SA and antibody against PCV2 and in order to avoid interference between domains, a flexible amino linker was used to link the two parts while keeping the domains apart. A flexible linker is critical in yielding the correct folding of the fusion protein. Experiments showed that binding affinity to HRP-SA of a fusion protein without linker was significantly lower, as was the reactivity with PCV2 antibodies (data not shown). Hsb-Cap∆41 is obtained by simply mixing SBP-Cap∆41 with HRP-SA and incubating at 4 °C. However, an incubation time of more than 48 h was necessary to ensure full binding between SBP-Cap∆41 and HRP-SA to permit optimal chromogenic reaction (Fig. 6). Since SA has four binding sites to biotin, HRP-SA was mixed with SBP-Cap∆41 in a molar ratio of 1:4. Addition of biotin prevented interaction with PCV2 antibodies, thereby confirming the specificity of the Hsb-Cap∆41 complex (Fig. 6).

The performance evaluation of the HBDS-ELISA indicated that it had high agreement with the DS-ELISA and the indirect ELISA. Moreover, longitudinal antibody profiles of the nine piglets naturally infected with PCV2 indicated that the sensitivity of the HBDS-ELISA was higher than the DS-ELISA since the S/P ratios of the positive sera were higher at most of the stages during the experimental period (Fig. 4). While the 4-week interval between samplings in this experiment was too long to permit evaluation of the sensitivity of the assay for antibodies elicited at the earliest stage of PCV2 infection, another set of serum samples collected between 0 and 16 days after injection with PCV2 indicated that, here also, the HBDS-ELISA was more sensitive than the DS-ELISA.

In conclusion, an Hsb-Ag (Hsb-Cap∆41) constructed by affinity binding between SBP fusion protein and HRP-SA can be used in a double-antigen sandwich ELISA. The Hsb-Ag based HBDS-ELISA described here showed higher diagnostic sensitivity for detection of PCV2 antibody than a chemical conjugated-antigen based DS-ELISA. The preparation procedure of Hsb-Ags is simple and could have wide application for the establishment of ELISAs for detection of antibodies to many other pathogens.

## Additional file

**Additional file 1: Text S1.** PCV2 CapΔ41 gene sequence.

## Abbreviations

PCV2: porcine circovirus 2
CSFV: classic swine fever virus
PRRSV: porcine reproductive and respiratory syndrome virus
PRV: porcine pseudorabies virus
PPV: porcine parvovirus
SBP: streptavidin binding peptide
HRP: horseradish peroxidase
SA: streptavidin
Hsb-Ag: HRP-streptavidin bound antigen
ELISA: enzyme linked immunosorbent assay
DS-ELISA: double-antigen sandwich ELISA
HBDS-ELISA: HSb-Cap∆41 based double-antigen sandwich ELISA
PCR: polymerase chain reaction
*E. coli*: *Escherichia coli*
SDS-PAGE: sodium dodecyl sulfate–polyacrylamide gel electrophoresis
IPTG: isopropyl-β-d-thiogalactoside
Trx: thioredoxin
PBST: phosphate-buffered saline containing 0.05% Tween 20
SD: standard deviation
CV: coefficient of variation
