# Supplementary material for: Construction of an HRP-streptavidin bound antigen and its application in an ELISA for porcine circovirus 2 antibodies
Source: AMB Express. 2017 Sep 18;7:177. doi: 10.1186/s13568-017-0473-3 (PMC5603472; doi:10.1186/s13568-017-0473-3)
Supplement: Supplementary file 1 — Additional file 1: Text S1. PCV2 CapΔ41 gene sequence. [file 13568_2017_473_MOESM1_ESM.docx]

Supplementary material

*AMB express*

Construction of an HRP-Streptavidin Bound Antigen and its Application in an ELISA for Porcine Circovirus 2 Antibodies

Meng Ge ^1, 2^, Run-Cheng Li ^2^, Tailong Qu ^2^, Wenjie Gong ^1^,·Xing-Long Yu ^2^*, Changchun Tu^1^*

*Correspondence: changchun_tu@hotmail.com; xlyu999@126.com.

^1^ Institute of Military Veterinary Medicine, Academy of Military Medical Sciences, No. 666 Liuying Xi Road, Changchun 130122, Jilin, People’ s Republic of China.

^2^ College of Veterinary Medicine, Hunan Agricultural University, No. 1 Nongda Road, Changsha, 410128, Hunan, People’ s Republic of China.

Text S1

PCV2 CapΔ41 gene sequence

AATGGCATCTTCAACACCCGCCTCTCCCGCACCATCGGTTATACTGTCAAAGCTACCACAGTAAGAACGCCCTCCTGGAATGTGGACATGATGAGATTTAATATTAATGATTTTCTTCCCCCAGGAGGGGGCTCAAACCCCCTCACTGTGCCCTTTGAATACTACAGAATAAGGAAGGTTAAGGTTGAATTCTGGCCCTGCTCCCCAATCACCCAGGGTGACAGGGGAGTGGGCTCCACTGCTGTTATTCTAGATGATAACTTTGTAACAAAGGCCAATGCCCTAACCTATGACCCCTATGTAAACTACTCCTCCCGCCATACCATACCCCAGCCCTTCTCCTACCACTCCCGCTATTTCACCCCCAAACCTGTCCTTGATAGGACAATCGATTACTTCCAACCCAATAACAAAAGAAATCAACTCTGGCTGAGACTACAAACTACTGGAAATGTAGACCATGTAGGCCTCGGCACTGCGTTCGAAAACAGTATATACGACCAGGACTACAATATCCGTATAACCATGTATGTACAATTCAGAGAATTTAATCTTAAAGACCCCCCACTTAACCCTAAGTGA
